# Numerical Analysis on Heat Transfer Characteristics of Supercritical CO_2_ in Heated Vertical Up-Flow Tube

**DOI:** 10.3390/ma13030723

**Published:** 2020-02-05

**Authors:** Chenshuai Yan, Jinliang Xu, Bingguo Zhu, Guanglin Liu

**Affiliations:** 1 The Beijing Key Laboratory of Multiphase Flow and Heat Transfer, North China Electric Power University, Beijing 102206, China; yanchenshuai@ncepu.edu.cn (C.Y.); zbg@ncepu.edu.cn (B.Z.); liu0513@ncepu.edu.cn (G.L.); 2 Key Laboratory of Power Station Energy Transfer Conversion and System, North China Electric Power University, Ministry of Education, Beijing 102206, China

**Keywords:** supercritical CO_2_, vertical tube, heat transfer mechanism, numerical analysis

## Abstract

It is great significance to understand the mechanism of heat transfer deterioration of supercritical CO_2_ for heat exchanger design and safe operation in the supercritical CO_2_ Brayton cycle. Three-dimensional steady-state numerical simulation was performed to investigate the behavior of supercritical CO_2_ heat transfer in heated vertical up-flow tube with inner diameter *d*_i_ = 10 mm and heated length *L*_h_ = 2000 mm. Based on the characteristics of inverted-annular film boiling at subcritical pressure, the heat transfer model of supercritical CO_2_ flowing in the heated vertical tube was established in this paper. The mechanisms of heat transfer deterioration (HTD) and heat transfer recovery (HTR) for supercritical CO_2_ were discussed. Numerical results demonstrate that HTD is affected by multiple factors, such as the thickness and property of vapor-like film near the wall, the turbulence intensity near the interface between liquid-like and vapor-like, and in the liquid-like core region as well as the distribution of radial velocity vector. Among the above factors, the change of turbulent kinetic energy caused by the buoyancy effect seems to be a more important contributor to HTD and HTR. Furthermore, the influences of heat flux and mass flux on the distribution of wall temperature were analyzed, respectively. The reasons for the difference in wall temperature at different heat fluxes and mass fluxes were explained by capturing detailed thermal physical properties and turbulence fields. The present investigation can provide valuable information for the design optimization and safe operation of a supercritical CO_2_ heat exchanger.

## 1. Introduction

Compared with the water–steam Rankine cycle, the supercritical CO_2_ Brayton cycle has been widely considered for power conversion systems in various energy industries, such as nuclear energy, fossil fuel, solar energy, and geothermal energy due to its high efficiency and system compactness. From the perspective of physical property, CO_2_ has relatively low critical parameters (critical pressure *P*_cr_ = 7.38 MPa, critical temperature *T*_cr_ = 304.25 K), and it is difficult to oxidize between CO_2_ and metal materials, which makes it possible to raise steam parameters including pressure and temperature for improving thermal efficiency [1]. Therefore, supercritical CO_2_ Brayton cycle has gradually become a research issue all over the world [2,3,4,5,6]. It is no doubt that heat transfer from heat source to supercritical CO_2_ is carried out through heat exchanger. Consequently, one of the key issues relating supercritical CO_2_ Brayton cycle is the safety of the heat exchanger. A comprehensive understanding of the heat transfer characteristic of supercritical CO_2_ flowing in a circular tube is essential to engineering design and the safe operation of heat exchangers.

In general, three heat transfer modes are divided during supercritical fluid flowing in heated tube: normal heat transfer (NHT), heat transfer enhancement (HTE), and heat transfer deterioration (HTD). In order to distinguish from HTD, researchers usually refer to NHT and HTE as non-HTD mode [7]. Compared with non-HTD mode, HTD is characterized by pronounced peaks with sharply increasing wall temperature or an abrupt decrease of heat transfer coefficient *h*, which will cause an obvious temperature difference between the heated surface and bulk fluid. Further, the heated surface may then overheat and fail. Therefore, HTD is more concerned during heat exchanger design and operation. Currently, many researchers have paid great attention to HTD of supercritical fluids, mainly establishing criterion to judge the onset of HTD for supercritical fluids [8,9,10,11,12] and revealing HTD mechanism.

For the HTD mechanism, numerous studies have mainly focused on the importance of flow acceleration effect and buoyancy effect. Jackson [13] proposed a criterion that was written as *Gr*/*Re*^2.7^ for predicting buoyancy effect on heat transfer. Jackson recommended that buoyancy effect could be ignored during supercritical fluids flowing in vertical tube when *Gr*/*Re*^2.7^ < 10^−5^, otherwise, it should be considered. Koshizuka et al. [14] numerically analyzed the HTD phenomenon of supercritical water. They considered that HTD was primarily triggered by the buoyancy effect at lower mass flux, and HTD appeared at the boundary between natural and forced convection. Fan et al. [15] presented that the main factor for HTD was the buoyancy effect at low mass flux and high heat flux, while the major reasons were not yet clear at high mass flux. Huang et al. [16] reviewed that both a strong flow acceleration effect and buoyancy effect were induced simultaneously at high heat flux, which caused complex flow and heat transfer behavior. Moreover, Huang et al. also summarized the criteria to judge the influence of buoyancy on heat transfer. They noted that there existed inconsistencies between the criteria and experimental data. They suggested that a new correlation was necessary to reliably predict the buoyancy effect. Liao and Zhao [17] experimentally investigated the convection heat transfer of supercritical CO_2_ in heated vertical miniature tubes, and found that buoyancy effect was still prominent at high mass flux. In conclusion, both the flow acceleration effect and buoyancy effect cannot thoroughly explain the HTD of supercritical fluids, and thus it is necessary to further investigate the heat transfer mechanism of supercritical CO_2_.

In the present research, the numerical investigation was conducted to reveal the heat transfer mechanism of supercritical CO_2_ in a vertical up-flow tube, and the effect of heat flux and mass flux on heat transfer was analyzed. The main content of this paper was organized as follows. Firstly, based on the characteristics of supercritical CO_2_ physical properties and previous studies, the physical heat transfer model of supercritical CO_2_ flowing in a vertical tube was established. Secondly, the heat transfer mechanism of supercritical CO_2_ was revealed according to the heat transfer model. Finally, the difference in supercritical CO_2_ heat transfer at different heat fluxes and mass fluxes were analyzed. The present investigation can provide the valuable information for optimization design and safe operation of heat exchanger.

## 2. Thermal-Physical Properties and Idealized Heat Transfer Model

**Figure 1:**
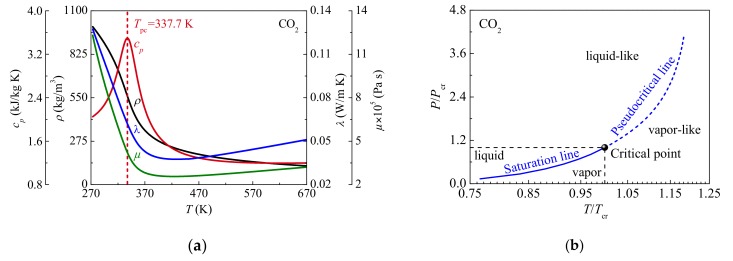
Thermal-physical properties and thermodynamic state plane of supercritical CO_2_: (**a**) The thermal-physical properties curves at *P* = 15.0 MPa; (**b**) The *P*/*P*_c__r_–*T*/*T*_c__r_ curves characterizing the transition between liquid and vapor at subcritical pressures and between liquid-like and vapor-like at supercritical pressures.

Figure 1a shows the thermal-physical properties (such as specific heat *c_p_*, density *ρ*, thermal conductivity λ and viscosity *μ*) of supercritical CO_2_ at *P* = 15.0 MPa. Different from the step change in properties at critical temperature under subcritical pressures, the thermal-physical properties of supercritical CO_2_ continuously change when temperature increases. As shown in Figure 1a, specific heat shows a peak at specified supercritical pressure. The temperature corresponding to maximum specific heat is defined as the pseudocritical temperature *T*_pc_. Figure 1b shows the *P*/*P*_cr_-*T*/*T*_cr_ curves at subcritical and supercritical pressures. According to classical thermodynamics, there exists no transition from liquid to vapor at supercritical pressures. However, the thermal-physical properties vary drastically and non-linearly within a narrow range of temperature near the pseudocritical temperature. Under supercritical pressures, the fluid behaves like vapor when its temperature is higher than *T*_pc_, and like liquid when its temperature is lower than *T*_pc_. The phenomenon is similar to the transition from liquid to vapor at subcritical pressures. Thus, for supercritical CO_2_, the fluid state is defined as liquid-like at *T* < *T*_pc_, and is defined as vapor-like at *T* > *T*_pc_. The pseudocritical temperature is regarded as the “pseudo-phase transition” temperature between liquid-like and vapor-like.

Holman et al. [18] visually analyzed forced convection heat transfer to supercritical Freon 12 in a vertical annulus and observed vapor trails. Holman et al. proposed that it seemed reasonable to name the phenomenon after “pseudo-boiling” even if there existed no liquid-vapor interface at supercritical temperature, because boiling was also caused by sharp density gradient at subcritical pressure. Wang et al. [19] concluded that the HTD of supercritical pressure water was mainly ascribed to low-density layer covering the heated surface from the macroscopic point of view. Zhang et al. [20] experimentally investigated the characteristics of supercritical CO_2_ heat transfer in heated vertical-flow tube, and mentioned that liquid-like near the tube wall transited to vapor-like when supercritical CO_2_ was gradually heated. They considered that the laminarization of vapor-like layer near the heated surface was a significant HTD mechanism.

**Figure 2:**
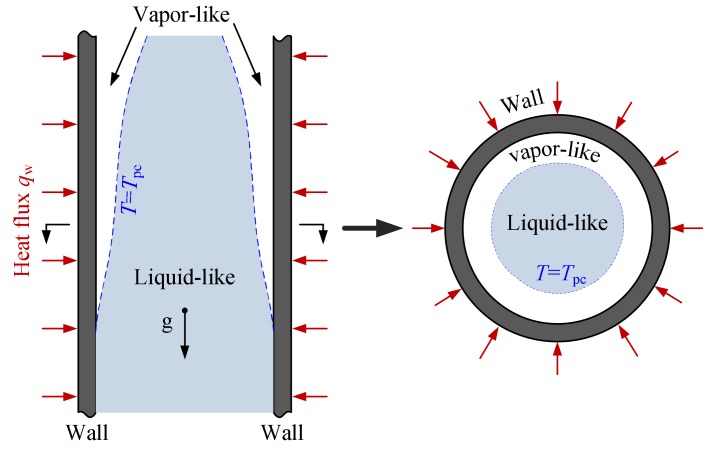
Schematic physical model of heat transfer for supercritical CO_2_ in present study.

Based on the above viewpoint, the physical heat transfer model of supercritical CO_2_ flowing in a heated vertical circular tube is established according to the characteristics of inverted-annular film boiling at subcritical pressure [21], which considers the effect of the low density fluid layer near the wall on heat transfer, as displayed in Figure 2. Under *T*_w,i_ > *T*_p,c_ > *T*_f_, low density fluid that is treated as vapor-like appears near the wall, and high density fluid that is treated as liquid-like flows in the center of the tube and is separated from the heated surface by vapor-like fluid, where *T*_w,i_ represents inner wall temperature and *T*_f_ represents local fluid temperature in the core region. The heat transfer process in the physical model mainly involves three steps: heat transfer from tube wall to vapor-like film, subsequently from vapor-like to interface between vapor-like and liquid-like, and then from the interface to liquid-like core.

In contrast to phase transition and the existence of a vapor-liquid interface at subcritical pressures, it should be noted that “pseudo-phase transition” process of supercritical CO_2_ near pseudocritical temperature is continuous, and the surface tension of supercritical CO_2_ is zero [22]. For the traditional numerical simulation of two-phase heat transfer at subcritical pressure, the surface tension of phase interface should be considered in the multiphase flow model [23,24]. Obviously, the multiphase flow model to investigate the boiling process at subcritical pressures is not suitable for supercritical fluids. Therefore, the numerical investigation of the heat transfer of supercritical CO_2_ still falls within the scope of single-phase flow and heat transfer.

## 3. Numerical Model and Method

### 3.1. The Governing Equations

The finite volume method is adopted to solve the transport equations of mass, momentum, and energy. The above governing equations are solved in a Cartesian coordinate system and can be expressed as below:

Mass

(1) $$\frac{\partial \left(\bar{\rho }{\tilde{u}}_{i}\right)}{\partial x_{i}}=0$$

Momentum

(2) $$\frac{\partial \left(\bar{\rho }{\tilde{u}}_{i}{\tilde{u}}_{j}\right)}{\partial x_{j}}=-\frac{\partial \bar{p}}{\partial x_{i}}+\frac{\partial }{\partial x_{j}}\left[\mu \left(\frac{\partial u_{i}}{\partial x_{j}}+\frac{\partial u_{j}}{\partial x_{i}}-\frac{2}{3}\delta_{ij}\frac{\partial u_{k}}{\partial x_{k}}\right)-\bar{\rho u_{i}^{’}u_{j}^{’}}\right]+\rho g_{i}$$

Energy

(3) $$\frac{\partial \left(\bar{\rho }{\tilde{u}}_{i}\tilde{h}\right)}{\partial x_{i}}=\frac{\partial }{\partial x_{i}}\left[\mu \left(\frac{1}{\mathrm{Pr}}+\frac{\mu_{t}/\mu }{{\mathrm{Pr}}_{t}}\right)\frac{\partial \tilde{h}}{\partial x_{i}}\right]$$

where superscript ‒ represents the time average scalar, superscript ~ represents the Favre average scalar, while *ρ*, *u*, λ and *h* are density, velocity, thermal conductivity, and fluid enthalpy, respectively.

For supercritical fluids, the turbulence model is great significant to the accuracy of calculation results. Liu et al. [25] and Wang et al. [26] discussed the abilities of different turbulence models to predict the experimental data of supercritical fluids, and found that shear stress transport (SST) *k*−*ω* low Reynolds model gave a more accurate prediction than other turbulence models. Consequently, the SST *k*−*ω* turbulence model is used in the current simulation.

The transport equations for SST *k*−*ω* model can be described as below:

Turbulent kinetic energy

(4) $$\frac{\partial \left(\bar{\rho }{\tilde{u}}_{i}k\right)}{\partial x_{i}}=\frac{\partial }{\partial x_{j}}\left[\left(\mu +\frac{\mu_{t}}{\sigma_{k}}\right)\frac{\partial k}{\partial x_{j}}\right]+G_{k}-Y_{k}$$

Specific dissipation rate

(5) $$\frac{\partial \left(\bar{\rho }{\tilde{u}}_{i}\omega \right)}{\partial x_{i}}=\frac{\partial }{\partial x_{j}}\left[\left(\mu +\frac{\mu_{t}}{\sigma_{\omega }}\right)\frac{\partial \omega }{\partial x_{j}}\right]+G_{\omega }-Y_{\omega }+D_{\omega }$$

*μ_t_* in Equations (4) and (5) is calculated by

(6) $$\mu_{t}=\frac{\rho k}{\omega }{\left[\max\left(\frac{1}{\alpha^{*}},\frac{F_{2}\sqrt{2S_{ij}S_{ij}}}{\alpha_{1}\omega }\right)\right]}^{-1}$$

where

α*=α∞*α0*+Ret/Rk1+Ret/Rk, $S_{ij}=\left(\frac{\partial u_{i}}{\partial x_{j}}+\frac{\partial u_{j}}{\partial x_{i}}\right)$

*G_k_*, *Y_k_*, *G_w_*, *Y_w_*, *D_w_* in Equations (4) and (5) are calculated by

(7) $$G_{k}=u_{t}\left(2S_{ij}S_{ij}\right)$$

(8) $$Y_{k}=\rho \beta^{*}k\omega$$

(9) Gω=α∞α*α0+Ret/Rω1+Ret/RωρμtGk

(10) $$Y_{\omega }=\rho \beta \omega^{2}$$

(11) $$D_{\omega }=2\left(1-F_{1}\right)\rho \sigma_{\omega ,2}\frac{1}{\omega }\frac{\partial k}{\partial x_{j}}\frac{\partial \omega }{\partial x_{j}}$$

In the above equations, the relevant constants can be referred in Ref. [27].

### 3.2. Physical Model and Gird Generation

**Figure 3:**
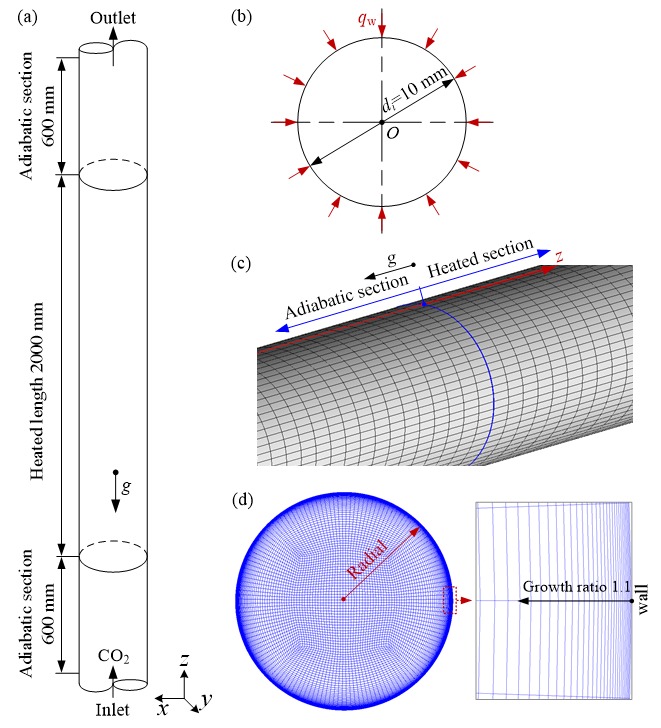
Physical model and computational grids adopted in the present simulation: (**a**) and (**b**) Physical model and coordinate system; (**c**) and (**d**) Grid generation in computational domain.

Figure 3a,b shows the physical model of a vertical tube with an inner diameter of 10 mm. The computational domain includes three parts: inlet adiabatic section, heated section and outlet adiabatic section. The heated length is 2000 mm. The length of inlet adiabatic section is 600 mm to ensure the fluid being fully developed before supercritical CO_2_ enters the heated section. The length of outlet adiabatic section is also 600 mm to ensure that supercritical CO_2_ flows steadily at outlet of heated section. The wall thickness of the simulated tube is ignored.

The structured grid technology is employed to generate hexahedron grid in the computational domain by ANSYS ICEM 15.0 software (ANSYS ICEM CFD 15.0,). Figure 3c,d show the grid generation in axial and radial direction, respectively. The axial grids are uniformly distributed. The O-type grid generation method is employed for a cross section of the tube. The grids near the wall-fluid interface are refined enough to accurately capture the dramatic variation of thermal-physical properties, and the non-dimensional wall distance *y*^+^ of the first node near the wall is always far less than 1 to meet the SST *k*−*ω* low Reynolds turbulence model requirement [28]. In the present study, the distance from the first node to the inner wall is set as 0.0015 mm and the growth ratio is set as 1.1.

### 3.3. The Boundary Conditions

The ANSYS Fluent 15.0 is adopted to perform numerical simulation for the flow and heat transfer of supercritical CO_2_ in vertical tube at three-dimensional steady state. The SIMPLEC algorithm is employed to solve the equations coupling pressure and velocity. The second-order upwind scheme is used to discretize the governing equations in Section 3.1, and the under-relaxation factors are set as default to achieve faster convergence. The maximum residual values are set as 1.0 × 10^−6^ for the mass and momentum equations and 1.0 × 10^−7^ for energy equation. The computation is assumed to converge when the iteration stops and the fluid temperature at outlet remains constant, and (|*q*_m,inlet_-*q*_m,outlet_|/*q*_m,inlet_) < 0.1%, where *q*_m_ represents mass flow rate at the same time. Wang et al. [29] analyzed the sensitivity of heat transfer to thermal-physical properties and considered that heat transfer coefficient was obviously affected by the thermal-physical properties. Thus, the NIST real gas model is employed and a dynamic connection will be formed between the REFPROP database and the Fluent solver. With each iteration, the REFPROP database will provide the corresponding thermal-physical properties to the Fluent solver, which can timely reflect the influence of drastic changes in thermal-physical properties on the characteristics of flow and heat transfer. Compared with the system pressure, the pressure drop along the tube can be neglected. Therefore, it is assumed that the system pressure is constant during flow and heat transfer [30]. The inlet and outlet boundary conditions are set as the mass-flow-inlet and pressure-outlet, respectively. The inner-wall surface is considered to be heated with a uniform and constant heat flux.

### 3.4. Grid Independence

In general, grid independence analysis should be performed to ensure the validity and accuracy of the simulated results. Table 1 shows seven grid systems for grid independence analysis. The influence of the number of nodes in axial and radial directions on numerical simulation is thoroughly considered in the current simulation.

**Figure 4:**
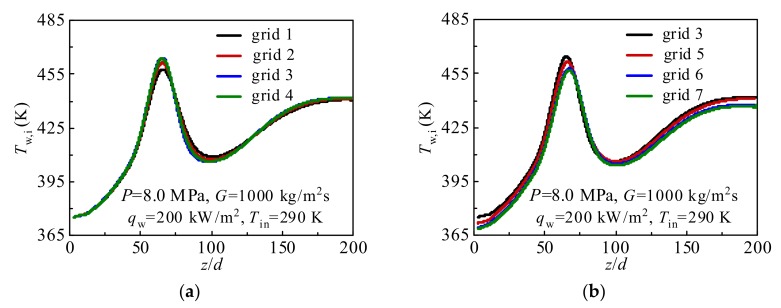
Validation of grid independence: (**a**) The influence of the number of axial nodes on the calculation results; (**b**) The influence of the number of radial nodes on the calculation results.

Figure 4 shows the results of grid independence analysis using seven grid systems under *P* = 8.0 MPa, *G* = 1000 kg/m^2^s, *q*_w_ = 200 kW/m^2^, *T*_in_ = 290 K. In Figure 4a, the Grid No. 4 is selected as the comparative item. It can be observed that there is no significant deviation in the calculated inner wall temperature *T*_w,i_ between Grid No. 3 and Grid No. 4. As shown in Figure 4b, the calculated inner wall temperature *T*_w,i_ between Grid No. 6 and Grid No. 7 are almost coincident when the result calculated by Grid No. 7 is selected as the standard. In addition, according to the Richardson extrapolation [31,32], the grid convergence index (GCI) is employed to further evaluate the grid quality. In general, it can be observed in Figure 4 that the calculation results are more sensitive to distributions of radial grids than that in axial direction. As a result, the coarse-grid, medium-grid, and fine-grid with grid numbers 1.13 × 10^6^ (Grid No. 3), 2.03 × 10^6^ (Grid No. 6), and 3.35 × 10^6^ (Grid No. 7) are selected for the GCI calculation.

GCI is defined as

(12) $$\mathrm{GCI}=\frac{F_{s}\cdot \varepsilon }{r^{p}-1}$$

where *F*_s_ represents the factor of safety and *F*_s_ = 1.25 [31], *ε* represents the relative error. *r* represents the ratio of grids number for the fine-grid and coarse-grid, *p* represents the formal order of algorithm accuracy, and the *ε*, *r*, and *p* can be expressed as [33]

(13) $$\varepsilon =\left|\frac{f_{1}-f_{2}}{f_{1}}\right|$$

(14) $$r={\left(\frac{N_{\mathrm{coarse}}}{N_{\mathrm{fine}}}\right)}^{\frac{1}{D}}$$

(15) $$p=\frac{\left|\ln\left|\left(f_{3}-f_{2}\right)/\left(f_{2}-f_{1}\right)\right|\right|}{\ln\left(r\right)}$$

where *f*_1_, *f*_2_, and *f*_3_ are the numerical solutions for fine-grid, medium-grid, and coarse-grid, respectively. *D* represents the geometry dimension. *N* is the number of control volumes. The value of GCI for the inner wall temperature at outlet is 0.037%. Therefore, Grid No. 6 with 2.03 × 10^6^ nodes is regarded as the optimal grid system in the subsequent numerical investigation in order to save computer resources and to simultaneously ensure the quality of the grid.

### 3.5. Model Validation

**Figure 5:**
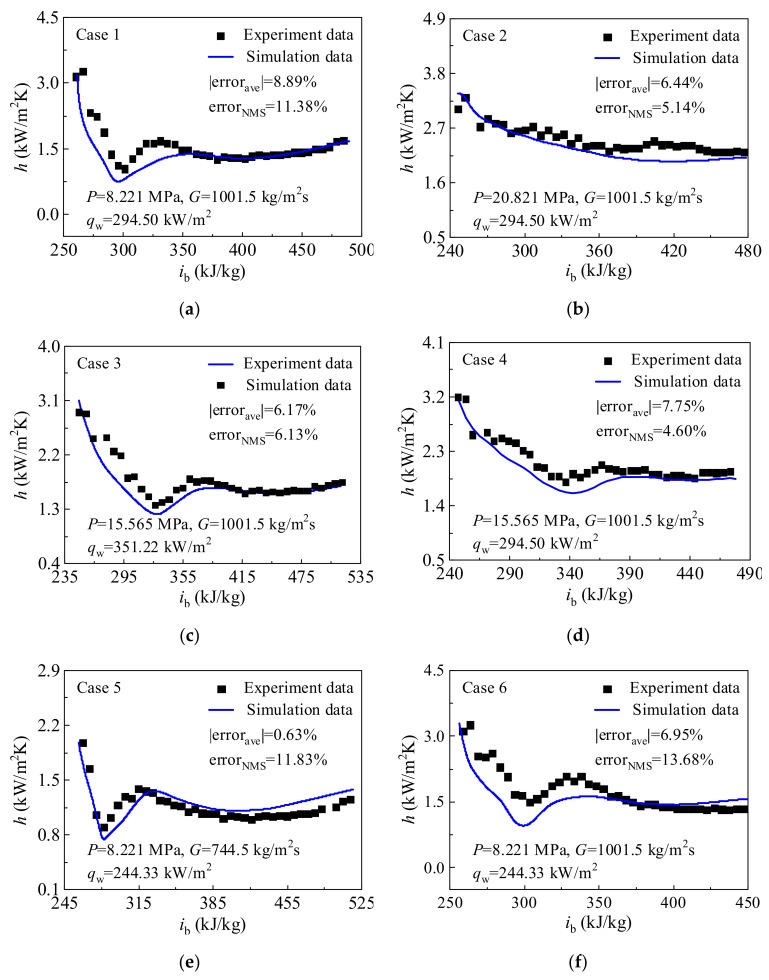
Comparison between predicted heat transfer coefficient *h* and experimental data. (**a**) *P*=8.221 MPa, *G*=1001.5 kg/m^2^s, *q*_w_=294.50 kW/m^2^; (**b)** *P*=20.821 MPa, *G*=1001.5 kg/m^2^s, *q*_w_=294.50 kW/m^2^; (**c**) *P*=15.565 MPa, *G*=1001.5 kg/m^2^s, *q*_w_=351.22 kW/m^2^; (**d**) *P*=15.565 MPa, *G*=1001.5 kg/m^2^s, *q*_w_=294.50 kW/m^2^; (**e**) *P*=8.221 MPa, *G*=744.5 kg/m^2^s, *q*_w_=244.33 kW/m^2^; (**f**) *P*=8.221 MPa, *G*=1001.5 kg/m^2^s, *q*_w_=244.33 kW/m^2^.

The experimental data from Ref. [12] are selected to validate the numerical model to ensure the accuracy of numerical simulation. The experimental data in Figure 5 are measured in Beijing Key Laboratory of Multiphase Flow and Heat Transfer. The experimental system, parameter measurements and uncertainties, as well as the date reduction method are detailed in Ref. [12], which are not repeated here. During the selection of experimental runs, the effects of operation pressure, heat flux, and mass flux on heat transfer are considered. The boundary conditions of experimental runs involved in this section are listed in Table 2, where inlet pressure *P*, mass flux *G*, heat flux *q*_w_, inlet temperature *T*_in_ and inlet Reynolds number *Re*_in_ are included. Figure 5a–f shows the comparisons between predicted heat transfer coefficient and experimental results. The variation trends of the predicted heat transfer coefficient show good consistency with the experimental data. Consequently, the numerical model is capable of simulating the flow and heat transfer of the supercritical CO_2_ in the present study.

In Figure 5, the mean relative error (error_ave_) and normalized mean square error (error_NMS_) between the predicted heat transfer coefficient *h*_pre_ and experimental *h*_exp_ are expressed as below:

(16) $${\mathrm{error}}_{\mathrm{ave}}=\frac{1}{n}\sum_{i=1}^{n}e_{i}\times 100\%$$

(17) $${\mathrm{error}}_{\mathrm{NMS}}=\sqrt{\frac{1}{n}\sum_{i=1}^{n}\left(e_{i}-\bar{e}\right)}\times 100\%$$

where *e_i_* represents the error for a single data point, $e_{i}=\left(h_{\mathrm{pre}}-h_{\exp}\right)/h_{\exp}$. $\bar{e}$ represents the mean relative error.

## 4. Results and Discussion

In the current simulation, *T*_b_ represents the bulk fluid temperature and is defined as [34]

(18) $$T_{b}=\int_{A}\rho uc_{p}TdA/\int_{A}\rho uc_{p}dA$$

where *A* is the cross-section area of the circular tube, m^2^, *ρ* represents the local fluid density, kg/m^3^. *u* represents the local fluid velocity, m/s, and *c_p_* represents the local fluid specific heat, kJ/kg K.

The bulk fluid enthalpy *i*_b_ can be defined as

(19) $$i_{b}=\int_{A}\rho uidA/\int_{A}\rho udA$$

where *i* represents the local fluid enthalpy, kJ/kg.

### 4.1. Mechanism of Heat Transfer Deterioration

**Figure 6:**
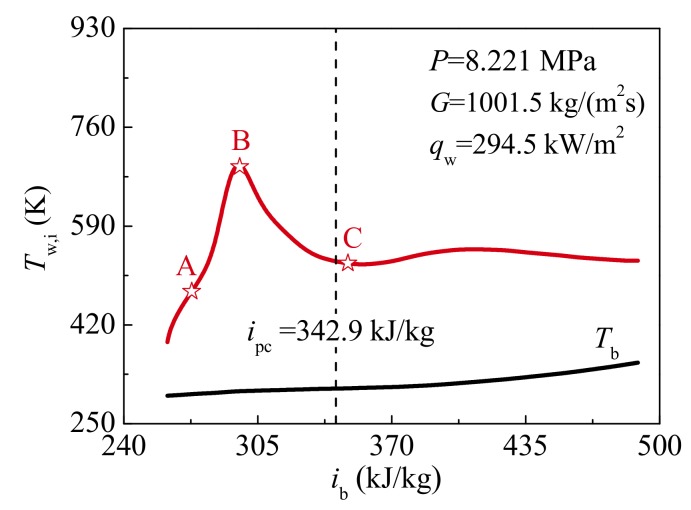
Variations of predicted inner wall temperature against bulk enthalphy at *P* = 8.221 MPa, *G* = 1001.5 kg/m^2^s and *q*_w_ = 294.50 kW/m^2^.

In this section, Case 1 in Table 2 is selected to analyze the heat transfer mechanism of supercritical CO_2_. Figure 6 shows the variations of predicted inner wall temperature *T*_w,i_ against bulk enthalphy *i*_b_ under *P* = 8.221 MPa, *G* = 1001.5 kg/m^2^s and *q*_w_ = 294.50 kW/m^2^. In Figure 5a and Figure 6, it can be found that the characteristic of wall temperature *T*_w,i_ and heat transfer coefficient *h* along the tube is consistent with HTD definition mentioned in the Introduction section. The characteristic locations A, B, and C are designated to reveal the mechanism of supercritical CO_2_ heat transfer, as shown in Figure 6. For convenient analysis, the HTD stage is defined from location A to B, and the HTR stage is defined from location B to C.

**Figure 7:**
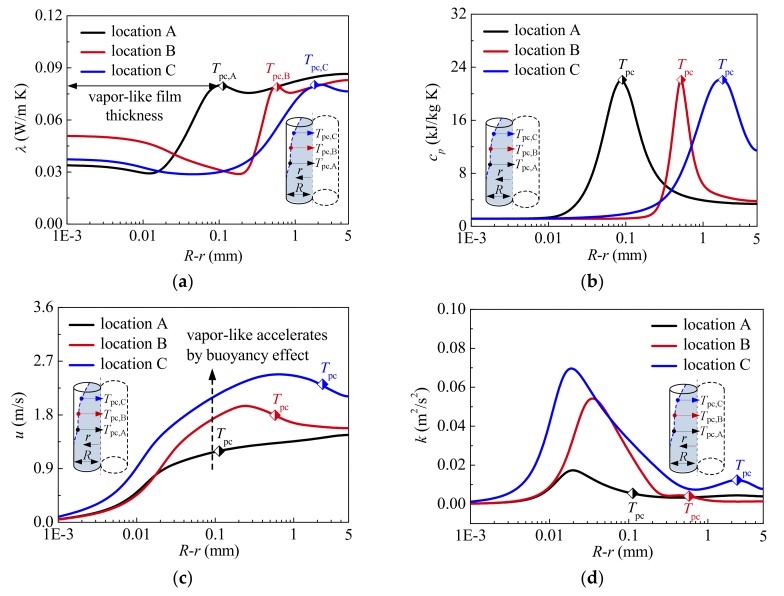
Thermal-physical properties and turbulence fields at characteristic locations A, B and C (*P* = 8.221 MPa, *G* = 1001.5 kg/m^2^s and *q*_w_ = 294.50 kW/m^2^): (**a**) Thermal conductivity; (**b**) Specific heat; (**c**) Axial velocity; (**d**) Turbulent kinetic energy.

Detailed information on the thermal-physical properties and turbulence fields at locations A, B, and C marked in Figure 6 is shown in Figure 7. It should be noted that *T*_pc_ is used to characterize the interface between liquid-like and vapor-like at steady state to evaluate the influence of vapor-like film on heat transfer. In other words, the distance from the location of *T*_pc_ to the tube wall indicates the vapor-like film thickness, as shown in Figure 7a. It is well known that the thicker the vapor-like film is, the farther large-property-variation region will deviate from tube wall. Further, the smaller the vapor-like film density is, the worse the film property characterized by thermal conductivity λ and specific heat *c_p_* is. Figure 7a,b shows that the thickness of vapor-like film gradually increases along the tube, and the thermal conductivity of vapor-like film generally decreases, which results in a large thermal resistance near the wall to prevent heat from the heated surface transferring to colder bulk fluid. The large specific heat region moves away from the tube wall at the same time, the ability of vapor-like film near the wall to absorb heat is weakened. The above phenomenon is very disadvantageous to the heat transfer of supercritical CO_2_. In other words, the thicker vapor-like film and the worse vapor-like property are the important reasons for HTD.

Figure 7c clearly depicts the radial distribution of axial velocity *u* at characteristic locations. Owing to the flow acceleration effect, the axial velocity of bulk fluid increases continuously. Especially, due to buoyancy effect, the axial velocity of vapor-like near the wall increases obviously and is deformed into M-shape, which has a momentous influence on the turbulence production. As shown in Figure 7d, because shear stress caused by the velocity gradient is gradually increased, the turbulent kinetic energy in vapor-like film increases generally along the tube. The characteristic can enhance heat transfer in vapor-like film near the wall. It should be noted that the change of turbulent kinetic energy in radial direction is mainly caused by local buoyancy effect near the wall.

Further, Figure 7d illustrates that the level of turbulent kinetic energy near the interface between liquid-like and vapor-like and in the liquid-like core region is gradually decreased at HTD stage, which means that the fluid near the vapor-liquid interface and in the core region approaches to laminarization. Owing to the inadequate momentum and heat diffusion, the heat passing through vapor-like film cannot be effectively transferred to the core region. “Heat transfer stagnation” almost occurs near vapor-liquid interface, and heat transfer is further seriously suppressed. As a result, the wall temperature rises sharply. At HTR stage, compared with the location B, although the vapor-like film is thicker at the location C, the turbulent kinetic energy near the vapor-liquid interface and in the core region is larger, which indicates the sufficient momentum and heat diffusion. Consequently, the heat from the heated surface can be effectively transferred to the liquid-like region, and then the wall temperature is decreased. Therefore, the ability of turbulence diffusion near the interface between the liquid-like and vapor-like, and in the liquid-like core region seems to be a more important reason contributing to HTD. Obviously, the mechanism of HTD analyzed by numerical simulation in the present investigation is different from that proposed in Ref. [20], where Zhang et al. considered that the laminarization of the vapor-like layer near the wall was an important HTD mechanism.

**Figure 8:**
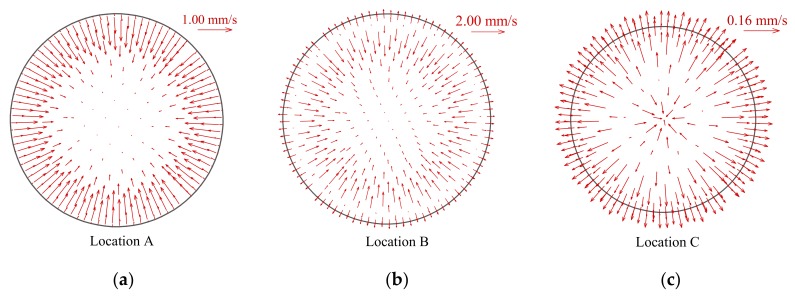
Distributions of radial velocity vector at characteristic locations A, B and C (*P* = 8.221 MPa, *G* = 1001.5 kg/m^2^s and *q*_w_ = 294.50 kW/m^2^). (**a**) location A; (**b**) location B; (**c**) location C.

It is interesting to notice that the HTD and HTR seem to be also related to the distribution of the radial velocity vector, as shown in Figure 8. At the HTD stage, the radial velocity near the wall is initially toward the center of tube cross section, which implies that the fluid in cross section has a tendency to flow toward the center of tube, as illustrated in Figure 8a. As a result, the tube wall cannot be effectively cooled in radial direction, the occurrence of HTD is accelerated. However, near the highest wall temperature at HTD stage, the radial velocity near the wall begins to turn, and then at HTR stage, the radial velocity vector towards the tube wall extends gradually to central region, as clearly shown in Figure 8b,c. This trend enhances mixing between the hot and cold fluid in cross section, and the tube wall can be cooled by the fluid in radial direction, which contributes to enhancing heat transfer and wall temperature recovery.

Therefore, the occurrence of HTD is caused by multi-factors including the thickness and property of vapor-like film near the wall, turbulent kinetic energy distribution affected by buoyancy as well as radial velocity vector. In other words, it is not only the buoyancy effect that leads to HTD.

### 4.2. The Effect of Heat Flux

**Figure 9:**
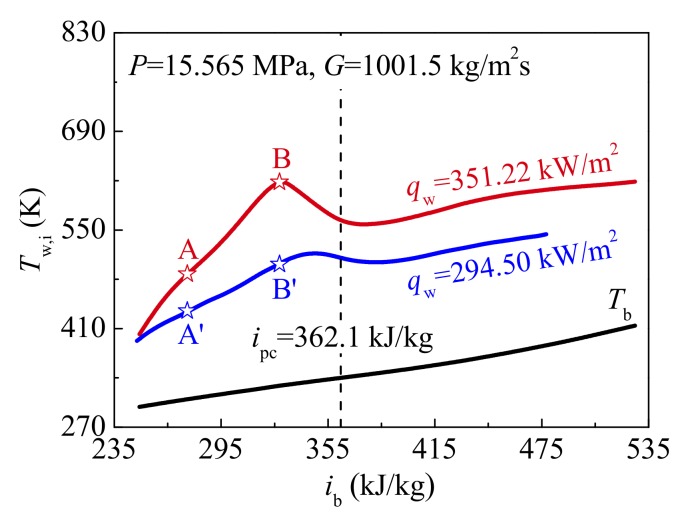
Variations of inner wall temperature against bulk enthalphy at different heat fluxes (*P* = 15.565 MPa, *G* = 1001.5 kg/m^2·^s).

Case 3 and Case 4 in Table 2 are selected to evaluate the effect of heat fluxes on the flow and heat transfer of supercritical CO_2_. The distribution curves of predicted inner wall temperature *T*_w,i_ against bulk enthalphy *i*_b_ at different heat fluxes are shown in Figure 9. At the relatively high heat flux (*q*_w_ = 351.22 kW/m^2^), the wall temperature is obviously increased, even HTD is more serious. Heat transfer is also deteriorated at the relatively low heat flux (*q*_w_ = 294.50 kW/m^2^) in present simulation, but *T*_w,i_ is gently changed. As shown in Figure 9, the characteristic locations A and A’ (*i*_b,A_ = *i*_b,A’_ = 276.0 kJ/kg), B and B’ (*i*_b,B_ = *i*_b,B’_ = 327.8 kJ/kg) are marked to analyze the reason for the difference in heat transfer of supercritical CO_2_ at different heat fluxes.

**Figure 10:**
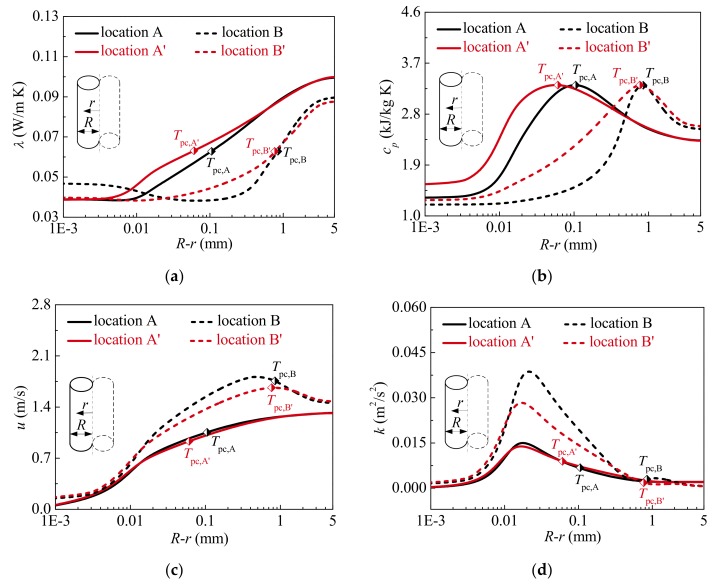
Thermal-physical properties and turbulent flow fields at characteristic locations (*P* = 15.565 MPa, *G* = 1001.5 kg/m^2^s): (**a**) Thermal conductivity; (**b**) Specific heat; (**c**) Axial velocity; (**d**) Turbulent kinetic energy.

The distributions of thermal-physical properties and turbulent flow at locations A and A’, B and B’ are presented in Figure 10. At high heat flux, the vapor-like film is thicker, and the property of vapor film characterized by specific heat and thermal conductivity is worse, as shown in Figure 10a,b. As a result, the ability of fluid near the wall to absorb and transfer heat is weakened. In other words, the thermal resistance caused by vapor-like film is larger at high heat flux. In addition, as can be found in Figure 10c,d, there is no obvious difference in the turbulent flow fields between location A and A’. However, compared with the location B’, due to a more significant buoyancy effect, the axial velocity *u* near the wall increases more obviously at location B. Therefore, the shear stress near the wall is increased, which is ultimately reflected by the distribution of turbulent kinetic energy, as displayed in Figure 10d. This implies that a change of turbulent flow near the wall can promote heat from the heated surface to pass through the vapor-like film at high heat flux. Comparing the locations B and B’, it should be noted that there is little difference in turbulent kinetic energy in the core region. 

Consequently, due to the thicker vapor-like film and the worse film property near the wall, the more heat cannot be removed in a timely manner from the heated surface at relatively high heat flux, which contributes to higher wall temperature and even more severe HTD.

**Figure 11:**
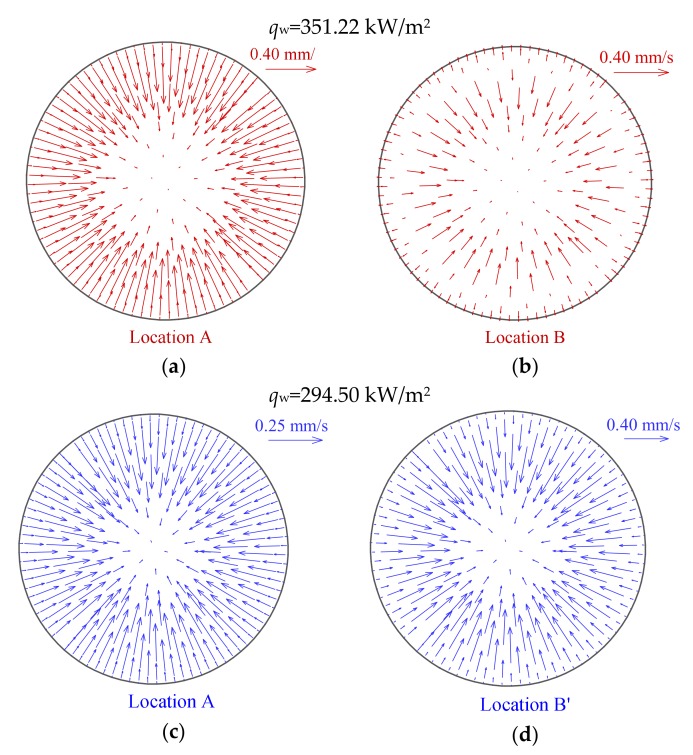
Distributions of radial velocity vector at characteristic locations at different heat fluxes (*P* = 15.565 MPa, *G* = 1001.5 kg/m^2^s): (**a**) and (**b**) *q*_w_ = 351.22 kW/m^2^; (**c**) and (**d**) *q*_w_ = 294.50 kW/m^2^.

In addition, Figure 11 presents the distributions of radial velocity vector at characteristic locations at different heat fluxes. The magnitude order of velocity between high and low heat fluxes is the same in present simulated case, which illustrates that the distribution of radial velocity vector has little effect on the difference in wall temperature at different heat fluxes. It is noteworthy that the distribution characteristics of radial velocity vector at HTD stages shown in Figure 11a,b are similar to those of Case 1 in Section 4.1. Thus, it is further demonstrated that the distribution of wall temperature along the tube is also related to the radial velocity vector at specified operation parameters during supercritical CO_2_ heat transfer.

### 4.3. The Effect of Mass Flux

**Figure 12:**
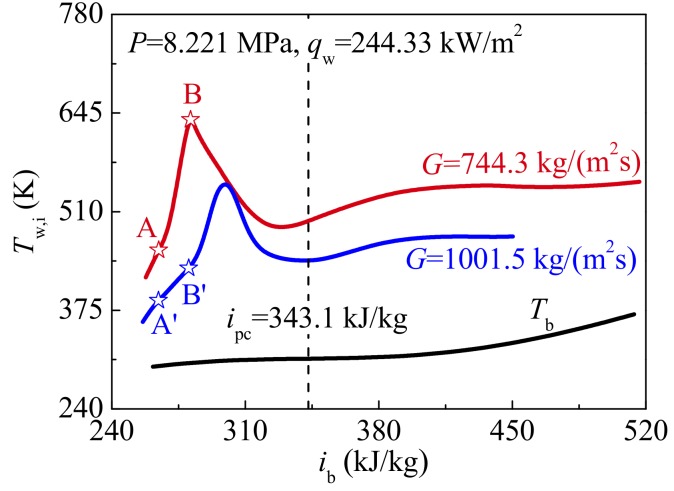
Variations of inner wall temperature against bulk enthalphy at different mass fluxes (*P* = 8.221 MPa, *q*_w_ = 244.33 kg/m^2^s).

Case 5 and Case 6 in Table 2 are selected to evaluate the effect of mass fluxes on heat transfer of supercritical CO_2_. The distribution curves of predicted inner wall temperature *T*_w,i_ against bulk enthalphy *i*_b_ at different mass fluxes are shown in Figure 12. It is observed that *T*_w,i_ increases as the mass flux decreases. In the present analysis, although the HTD occurs at both high and low mass fluxes, the amplitude of wall temperature overshoot is smaller at the relatively high mass flux. It illustrates that high mass flux can suppress HTD occurrence. The characteristic locations A and A’ (*i*_b,A_ = *i*_b,A’_ = 264.6 kJ/kg), B and B’ (*i*_b,B_ = *i*_b,B’_ = 280.3 kJ/kg) are marked to analyze the reason for difference in heat transfer of supercritical CO_2_ at different mass fluxes, as shown in Figure 12.

**Figure 13:**
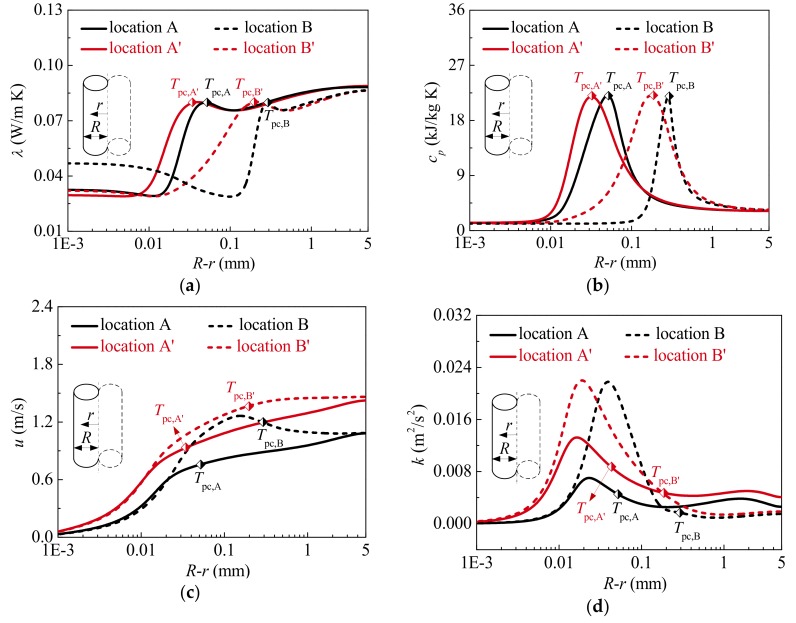
Thermal-physical properties and turbulent flow fields at characteristic locations (*P* = 8.221 MPa, *q*_w_ = 244.33 kg/m^2^s): (**a**) Thermal conductivity; (**b**) Specific heat; (**c**) Axial velocity; (**d**) Turbulent kinetic energy.

Figure 13 shows the distributions of thermal-physical properties and turbulent flow at the locations A and A’, B and B’ marked in Figure 12. At low mass flux, the vapor-like film is thicker, and its property is worse, as shown in Figure 13a,b. This illustrates that the thermal resistance of vapor-like film covering the heated surface is larger at low mass flux. Simultaneously, at low mass flux, the lower axial velocity cannot timely remove heat from the heated surface, and the turbulent kinetic energy is also generally smaller, especially in the liquid-like core region, which weakens heat transfer in the radial direction, as shown in Figure 13c,d.

**Figure 14:**
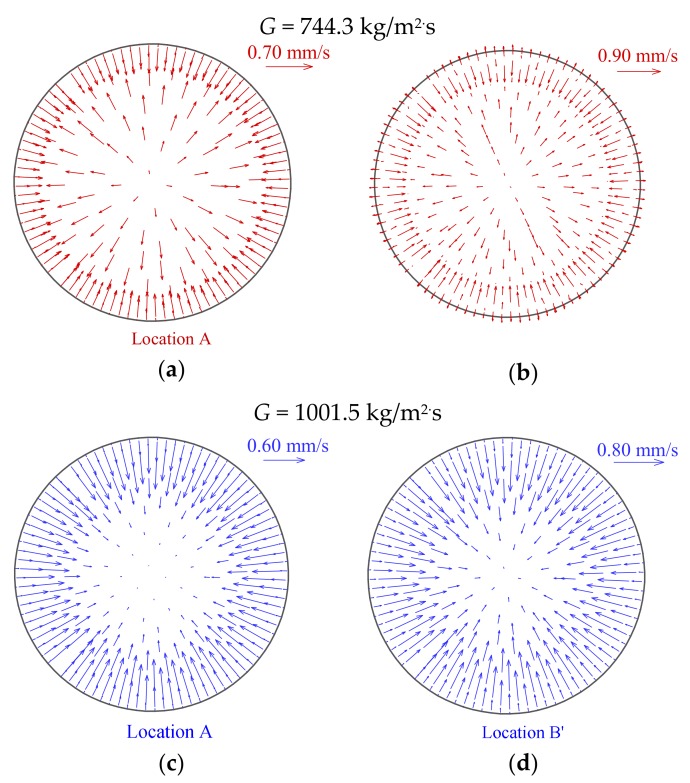
Distributions of radial velocity vector at characteristic locations at different mass fluxes (*P* = 8.221 MPa, *q*_w_ = 244.33 kg/m^2^s): (**a**) and (**b**) *G* = 744.3 kg/m^2^s; (**c**) and (**d**) *G* = 1001.5 kg/m^2^s.

Figure 14 shows the distributions of radial velocity vector at characteristic locations under different mass fluxes. It is of interest to see that, in contrast to the distribution of radial velocity vector at high mass flux, the velocity vector direction of supercritical CO_2_ in the core region is always toward the tube wall at low mass flux, as shown in Figure 14a,b. However, at different mass fluxes, there is a common ground that the velocity vector near the wall is always toward the center of tube at the beginning of the HTD stage. At low mass flux, it should be noted that velocity vector also begins to turn near the highest wall temperature at HTD stage, as shown in Figure 14b. This conclusion that the radial velocity vector affects the heat transfer of supercritical CO_2_ is consistent with Section 4.1 and Section 4.2.

## 5. Conclusions

In this paper, the numerical analysis was performed to investigate the flow and heat transfer characteristics of supercritical CO_2_ in heated vertical up-flow tube. The mechanisms of HTD and HTR for supercritical CO_2_ were revealed. The reasons for the difference in wall temperature distribution at different heat fluxes and mass fluxes were analyzed. Based on the present study, the main conclusions are presented as follows:

- The occurrence of HTD is ascribed to the comprehensive effects of multiple factors. The vapor-like film with a large thermal resistance covering the heated surface prevents heat from the tube wall from transferring to the cooler bulk fluid. Simultaneously, it is another important reason for HTD that the fluid near the interface between vapor-like and liquid-like, and in the core region gradually approaches laminarization. Due to inadequate momentum and heat diffusion, heat transfer in the core fluid is seriously suppressed. Further, the radial velocity vector of fluid near the wall is initially toward the center of the tube, which results in the heated surface failing to be cooled effectively by the fluid in radial direction.
- The formation of HTR is mainly attributed to two factors. The increased turbulent kinetic energy promotes heat transfer in radial direction, especially near the interface between liquid-like and vapor-like and in the liquid-like core region. The radial velocity vector of supercritical CO_2_ is towards the tube wall at HTR stage, thus the tube wall can be cooled by the fluid in radial direction.
- For the relatively high heat flux, owing to the thicker vapor-like film and the worse film property near the wall, the more heat cannot be removed timely from the heated wall, which results in a higher wall temperature and even more serious HTD. For the relatively low mass flux, the vapor-like film is thicker, and its property is worse, and the lower axial velocity and the level of turbulent kinetic energy weaken heat transfer in axial and radial directions, which result in HTD being more likely to occur.

## Figures and Tables

**Table 1:** Cases for grid independence analysis.

Grid No.	Number of Nodes		Total Number of Nodes
	Radial	Axial	
1	44	100	0.29 × 10^6^
2	44	230	0.67 × 10^6^
3	44	390	1.13 × 10^6^
4	44	480	1.39 × 10^6^
5	50	390	1.44 × 10^6^
6	65	390	2.03 × 10^6^
7	75	390	3.35 × 10^6^

**Table 2:** Boundary conditions of experiment runs for model validation [12].

Case No.	*P* (MPa)	*G* (kg/m^2^s)	*q*_w_ (kW/m^2^)	*T*_in_ (K)	*Re*_in_
1	8.221	1001.5	294.50	298.0	1.473 × 10^5^
2	20.821	1001.5	294.50	299.1	1.051 × 10^5^
3	15.565	1001.5	351.22	298.0	1.148 × 10^5^
4	15.565	1001.5	294.50	295.8	1.112 × 10^5^
5	8.221	744.5	244.33	296.5	1.053 × 10^5^
6	8.221	1001.5	244.33	296.5	1.417 × 10^5^
